# Evaluation of novel cationic gene based liposomes with cyclodextrin prepared by thin film hydration and microfluidic systems

**DOI:** 10.1038/s41598-019-51065-4

**Published:** 2019-10-22

**Authors:** Hassan Elsana, Temidayo O. B. Olusanya, Jane Carr-wilkinson, Steven Darby, Ahmed Faheem, Amal Ali Elkordy

**Affiliations:** University of Sunderland, School of Pharmacy and Pharmaceutical Sciences, Sunderland, SR1 3SD UK

**Keywords:** Drug discovery, Medical research

## Abstract

In gene delivery, non-viral vectors have become the preferred carrier system for DNA delivery. They can overcome major viral issues such as immunogenicity and mutagenicity. Cationic lipid-mediated gene transfer is one of the most commonly used non-viral vectors, which have been shown to be a safe and effective carrier. However, their use in gene delivery often exhibits low transfection efficiency and stability. The aim of this study was to examine the effectiveness of novel non-viral gene delivery systems. This study has investigated the encapsulation and transfection efficiency of cationic liposomes prepared from DOTAP and carboxymethyl-β-cyclodextrin (CD). The encapsulation efficiency of the CD-lipoplex complexes were also studied with and without the addition of Pluronic-F127, using both microfluidic and thin film hydration methods. *In vitro* transfection efficiencies of these complexes were determined in COS7 and SH-SY5Y cell lines. Formulation stability was evaluated using liposomes size, zeta potential and polydispersity index. In addition, the external morphology was studied using transmission electron microcopy (TEM). Results revealed that formulations produced by microfluidic method had smaller, more uniform and homogenious size and zeta-potential as well as higher encapsulation efficiency when compared with liposomes manufactured by thin film hydration method. Overall, the results of this study show that carboxymethyl-β-cyclodextrin increased lipoplexes’ encapsulation efficiency using both NanoAssemblr and rotary evaporator manufacturing processes. However, this increase was reduced slightly following the addition of Pluronic-F127. The addition of carboxymethyl-β-cyclodextrin to cationic liposomes resulted in an increase in transfection efficiency in mammalian cell lines. However, this increase appeared to be cell line specific, COS7 showed higher transfection efficiency compared to SH-SY5Y.

## Introduction

Liposomes are a form of spherical vesicles that consist of either one or many phospholipid bilayers that interacting in an energetically favourable way. Generally, liposomes are vesicles of self-assembled phospholipid molecules. Those molecules composed of hydrophilic head groups (typically tertiary or quaternary amino group) attached to hydrophobic tails (generally long-chain fatty acids) by a linker^1–3^. The amphiphilic nature of these lipid molecules causes them to form bilayers spontaneously in aqueous environments. This results in a small spherical structure in which the surface polar heads shield the non-polar interior against water. The positively charged amine groups enhance binding with negative groups in DNA for example^4^. Moreover, liposomes are attractive as a gene vector due to their ability to carry DNA to various target cells. In addition, liposome formulations have been established to be a safe carrier, with such formulations being used worldwide in different therapeutic and vaccinology products. Liposomes have also been used as a drug carrier to control drug delivery to protect the drug payload from rapid degradation, to enhance drug concentration in targeted tissues and to lower doses of the required drug and hence lowering toxicity. The versatile structure and low immunogenicity of liposomes have been shown to be a promising gene transfer system. Liposomes can entrap different molecules such as nucleic acids and may even protect DNA against enzymatic degradation within the cell. Liposomes also can enhance cellular uptake, endosomal escape and gene transfection. However, their application in gene therapy is hampered by the low transfection efficiency.

Liposomes can be classified according to their structure, size, the method of preparation and composition, these parameters will depend on the liposome application and type of molecule required to be encapsulated. Different methods for liposome preparation at the laboratory scale have been developed and optimised. These methods include hydration of phospholipid film (e.g. Al-Rubaie *et al*.^5^), reverse phase evaporation method, ether or ethanol injection (solvent vaporization) and microfluidics (see for example Obeid *et al*.^6^). The vesicle size will determine the circulation half life of liposomes and both size and number of bilayers affect the amount of encapsulated drug in liposomes. The circulation time of cationic liposomes in blood is short due to their surface charge. The systemic delivery of cationic lipoplexes to its target target site can also be affected by the interaction between serum and lipoplexes, which can dramatically lower the transfection efficiency^7^. The composition and methods of preparation can greatly influence the liposome basic properties. Which may in turn affect the polydispersity index, mean size, drug loading efficiency, zeta potential, drug release behavior, and intracellular uptake.

The aim of this research is to improve cationic liposome transfection efficiency and stability by incorporating carboxymethyl-β-cyclodextrin and Pluronic F-127 with cationic lipid DOTAP. Additionally, the liposome preparation methods of thin film hydration and microfluidics will be examined to determine if preparation methodology has a direct effect on transfection efficiency.

The rational behind the use of carboxymethyl-β-cyclodextrin and Pluronic F-127 is that β-CD is considered to have a wide variety of applications due to their large cavity, low cost and lack of toxicity^8^. The availability of many hydroxyl groups in CM-β-CD can form different types of linkages. Cyclodextrin has been shown to be a facile and practical approach in developing gene delivery when used as a core system or alongside cationic lipids^9^. The enhancement of gene transfection when liposomes are incorporated with less polar cyclodextrin could be a result of non-specific interactions with cell membrane ingestion by endocytosis rather than an electrostatic interaction^10–13^. Also, cycloextrin was included in this study, as cyclodextrins have prospective endosomal disrupting effects. These effects might be through the release of membrane components from endosomal membranes after endocytosis Incorporation of CD with an excipient such as folic acid or Pluronic-F127 can positively affect the stability and effectiveness of gene formulations.

To the best of our knowledge, there have been no previous studies that investigated the incorporation of carboxymethyl-β-cyclodextrin and Pluronic F-127 with the cationic lipid (DOTAP) in gene delivery.

## Results and Discussion

DNA condensation is a prerequisite for successful gene delivery. Carboxymethyl-β-cyclodextrin has shown to be effective in gene delivery and our previous study^14^ demonstrated that carboxymethyl-β-cyclodextrin has the ability to condense DNA at a molar ratio of 1:3 with 22% encapsulation efficiency. To investigate this further carboxymethyl-β-cyclodextrin (CD) was incorporated with cationic lipid (DOTAP), netural lipid (DOPE) and cholesterol to form liposomes to study the effect on gene cationic liposomes transfection and to evaluate the degree of gene encapsulation efficiency.

### Liposomes size, zeta potential, poly dispersity (pdi) and morphology

The disappearance of liposomes from blood circulation is primarily due to uptake of the liposomes by the mononuclear phagocytic system. A decrease in liposome size reduces complement recognition when the liposome size is between 70 and 200 nm^15,16^. Several articles published recently have also suggested that the particle size of gene delivery system should not exceed 150 nm. A recent study reported that 135 nm is the optimal size for gene delivery. This current study looked at the effect of liposome preparation methods and liposomes composition on liposome size. Microfluidic method using the NanoAssemblr^TM^ and the hydration method using the rotary evaporator were used to study these methods. In order to optimise results of this study, the NanoAssemblr was run at 12 ml/min, 9 ml/min, 5 ml/min and 2 ml/min and ratio at 1:0.5, 1:1,1:3 and 1:5 of organic:aqueous. During optimisation a significant advantage was observed with the use of NanoAssemblr over the rotary evaporator, this was due to it’s the NanoAssemblr’s ability to control liposomes size using flow rate, TFR, and flow rate ratio(FRR). It was observed that liposome size was changed when changing from 12 ml/min to 2 ml/min and when changing flow ratio from 1:1 to 1:5 (data not included). Statistical analysis of TFR and FRR results show that the reduction in liposomes size resulted from the change in TFR was not significantly different (p > 0.05) and the change in FRR was only significant between ratio 1:1 and 1:3 or 1:1 and 1:5 and there is no significant difference in between ratio 1:3 and 1:5. The rationale behind choosing ratio 1:3 over 1:1, at 1:3 liposomes will have less cationic lipid, and since high cationic lipid is associated with an increase in cell toxicity 1:3 ratio would be the better choice.This gives a control of liposomes size and hence no need for after production size reduction. However, zeta potential was only affected by change in flow ratio but not by total flow rate. These results confirmed that the nanoAssemblr at flow rate of 2 ml/min and at ratio of 1:3 lipid to aqueous, was able to produce size of less than 165 nm, with homogeneous size distribution. Therefore, these parameters were used to prepare liposomes for further studies such as gene encapsulation efficiency and cell transfection. Also, the NanoAssemblr had the added benefit of a a one-step preparation procedure and also had a shorter preparation time.

The sizes of liposomes prepared by the NanoAssemblr and the rotary evaporator can be seen in Table 1. Liposomes prepared by NanoAssemblr sizes were between 79.51 nm and 161 nm and between 109 nm and 294 nm for the rotary evaporator. These results are consistent with other studies^17–20^ that outline that the rotary evaporator method needs to be followed by ∼20 min sonication, yet size still between 109 and 294 nm. This study has also shown that liposomes composition has major effect on liposomes size.

**Table 1:** Particle size, PDI, Zeta potential(ζ) for formulations prepared by NanoAssemblr and rotary evaporator.

FORMULATION NUMBER	NanoAssemblr			Rotary Evaporator		
	Size nm	PDI	Zeta potential(ζ) mV	SIZE Nm	PDI	Zeta potential(ζ) mV
F1	79.51 ± 12	0.108 ± 0.009	44.1 ± 3	247 ± 40	0.324 ± 0.023	56 ± 6
F2	152 ± 32	0.126 ± 0.003	42.1 ± 3	109 ± 28	0.339 ± 0.017	48 ± 4
F3	95.71 ± 25	0.324 ± 0.012	27.6 ± 5	287 ± 31	0.336 ± 0.023	52 ± 6
F4	161 ± 21	0.194 ± 0.003	24.5 ± 4	186 ± 33	0.235 ± 0.002	45 ± 3
F5	105 ± 19	0.249 ± 0.019	35.8 ± 5	294 ± 29	0.303 ± 0.009	55 ± 2
F6	149 ± 20	0. 455 ± 0.03	34.1 ± 3	204 ± 23	0.341 ± 0.012	50.6 ± 4
F7	86.66 ± 32	0.127 ± 20	31.6 ± 6	217 ± 31	0.436 ± 0.013	53.5 ± 4
F8	98.27 ± 14	0.136 ± 0.013	29.4 ± 4	119 ± 28	0.239 ± 0.017	50.2 ± 6

Refer to Table 3 (in experimental section) for formulations’ composition.

The addition of carboxymethyl-β-cyclodextrin or carboxymethyl-β-cyclodextrin and Pluronic F-127 (Table 1) resulted in an increase in liposomes size. This increase in size could be due to cyclodextrin displacing cholesterol in the liposome formulation, which decreases liposome rigidity and increase its size^21^. Results in Table 1 have also shown an increase in size of liposomes (F1, F3, F5, F7) when DNA is incorporated (lipoplexes, F2, F4, F6, F8), which is in agreement with other published research^22^.

The Polydispersity Index (PDI) or particle distribution has a significant impact on liposomes stability and bioavailability. For liposomes to be stable, safe, and efficient liposomes preparation must be homogenous. An acceptable liposomes formulation for drug delivery should have PDI value below 0.3^23,24^. The liposomes prepared by the NanoAssemblr met this criteria, it was observed that many liposomes prepared by the rotary evaporator method were above this value (Table 1).

A positive zeta-potential not only has the benefit for enhancing pDNA loading efficiency but also for improving the effective accumulation in the target cells. A positive value will also impact liposomes stability, many studies have shown that ζ-potential values that range between +16 and +55 mV are high enough to ensure colloidal stability due to the electrostatic repulsion between particles.

Zeta potential(ζ) results (Table 1) revealed that all formulations were positively charged, before and after the addition of pDNA. Zeta potential values of lipoplexes were similar to the values obtained from liposomes alone, however there was a reduction in the zeta potential following the addition of pDNA. This could be a result of the electrostatic interaction between the cationic lipid and the negatively charged backbone of the pDNA^21^. Different zeta potential reading was reported for formulations prepared by each method (NanoAssemblr or rotary evaporator). NanoAssemblr showed lower zeta potential between 24.5 mv and 44.1 mv, whereas the rotary evaporator showed higher zeta value between 45 mv and 56 mv (Table 1). This can be explained by formulation homogenous suspensions achieved by the NanoAssemblr resulting in majority of the amino group been occupied which induce protonation on the surface of liposomes.

**Figure 1:**
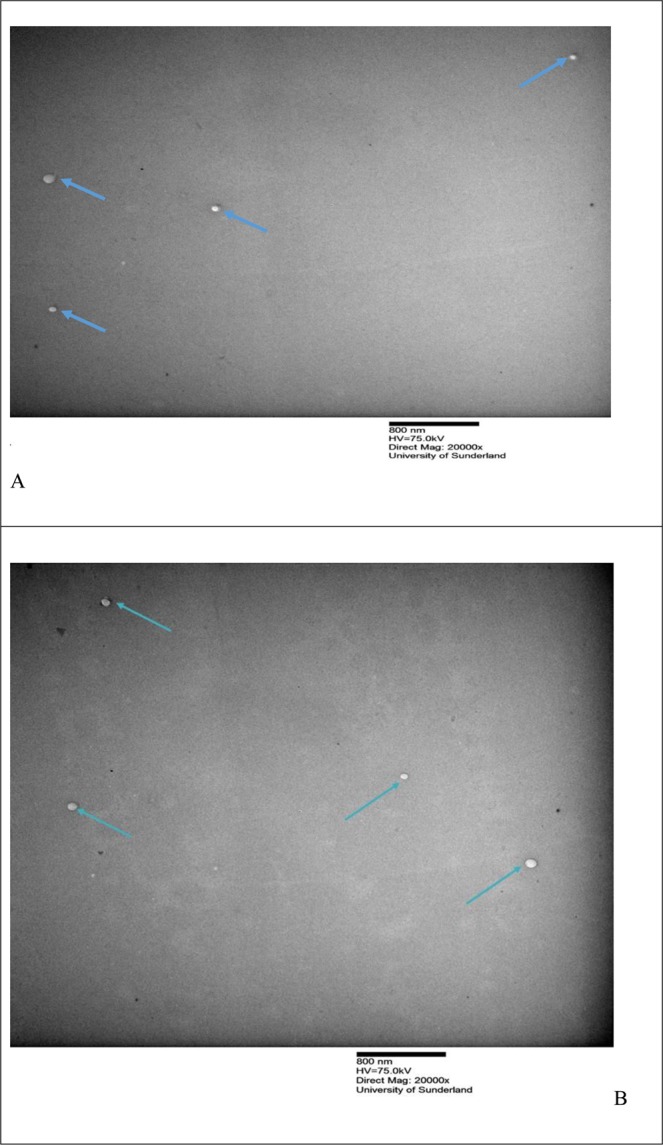
shows TEM images for different liposomes formulation using the nanoAssemblr. (**A**) (F1), (**B**) (F3). For formulation composition refer to Table 3 (in experimental section).

**Figure 2:**
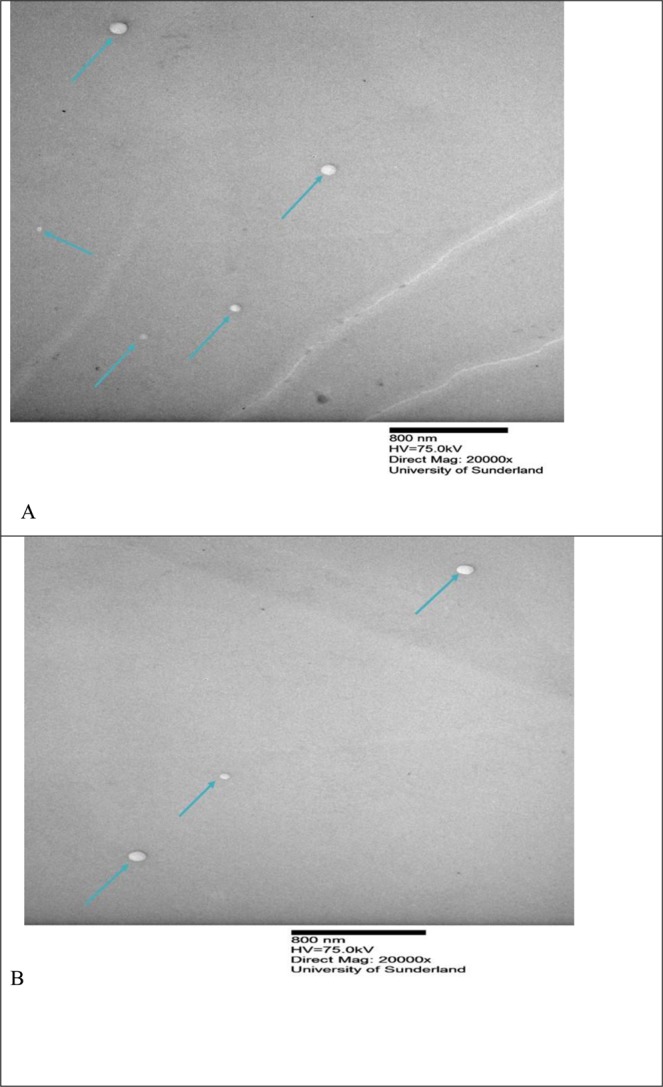
shows the images of different liposomes formulations using the rotary evaporator. (**A**) (F1), (**B**) (F3). For formulation composition refer to Table 3 (in experimental section).

Transmission Electron Microscopy (TEM) was used to investigate the external of the liposomes morphology. Morphology of fresh liposomes prepared by NanoAssemblr and rotary evaporator are illustrated in Figs 1 and 2. TEM images were taken at the same magnification of 40000x for fresh liposomes with and without cyclodextrin. All images showed clear spherical shape of liposomes, with unilamellar as well as very small number of multilamellar liposomes observed in some samples. No change in liposomes morphology was observed after the addition of cyclodextrin.

### Encapsulation efficiency

Previous studies have shown that the addition of β-cyclodextrin to liposomes has resulted in increase of encapsulation efficiency of different small drug molecules (e.g.^25,26^). However, the role of cyclodextrin-liposome complex in gene therapy has not been characterised.

The encapsulation efficiency of pDNA was investigated in this current study by incorporating carboxymethyl-β-cyclodextrin with cationic liposomes. The percentage of encapsulation efficiency was calculated using Eq. 1. Results displayed in Table 2 revealed that all liposomes formulations prepared by both NanoAssemblr and rotary evaporator methods were able to encapsulate pDNA up to 89.6%.

**Table 2:** Encapsulation efficiency (EE) of pDNA inside liposomes using the microfluidic via nanoassemblr and thin film hydration, using rotary evaporator, methods.

Formulation	Samples (1:5 pDNA:liposome)	NanoAssemblr		Rotary Evaporator	
		EE	SD	EE	SD
F2	DOTAP + DOPE + CHO + p**DNA**	74.10%	0.66	66.95%	0.03
F4	DOTAP + DOPE + CHO + CD + p**DNA**	89.62%	0.52	75.93%	0.05
F6	DOTAP + DOPE + CHO + CD + PL + p**DNA**	76.50%	0.50	57.43%	0.08
F8	DOTAP + DOPE + CHO + PL + p**DNA**	78.30%	0.20	54.40%	0.80

The addition of carboxymethyl-β-cyclodextrin to cationic lipoplexes, F4, has resulted in increase in the encapsulation efficiency by 15% and 9% using the nanoAssemblr and rotary evaporator respectively compared to lipoplexes without (F2), (Table 2). This increase can be explained by the attraction of the electrostatic binding between the phosphate group of the DNA backbone and the lipophilic inner cavity of the cyclodextrin and the amino group in cationic liposomes. The increase in encapsulation efficiency after the addition of carboxymethyl-β-cyclodextrin to cationic liposomes in gene delivery has not been reported previously. Samples containing cyclodextrin and pluronic F-127 (F6) showed lower encapsulation efficiency than formulation F4, but still slightly better compared with formulation F2. This can be explained by that the addition of Pluronic F-127 would compete with pDNA for the cavity of cyclodextrin, resulting in a reduction of plasmid DNA entrapment confirmed with Pluronic F-127 in F8 lipoplexes.

The effectiveness of the microfluidic method was recently outlined in the encapsulation of pDNA for the transfection of COS 7 cells^20^. However, there was no direct comparison between the two methods. Thus, the results of this study allowed direct comparison and outlines an advantage of liposome preparation using microfluidic method rather than thin film hydration process. The improvement in the encapsulation efficiency using microfluidic method could be a result of the production of homogeneous liposomes formulations, and also to the presence of ethanol in liposomes formulations that can also enhance the encapsulation efficiency by making the lipid membrane susceptible to structural rearrangements^27^.

### Gel electrophoresis

DNA migrates through an agarose gel matrix by the action of an electric field according to its charge, size, and morphology. pDNA must survive in either supercoiled or open circular form in order to retain optimal gene expression, detection of double strand DNA is not enough to determine if the pDNA still in its active form. Liposomes and liposplexes were prepared as outlined in the methods section. All formulations were centrifuged for 45 min then re-suspended in distilled water. The use of centrifugation and DNase I will be an indication of where pDNA condensed inside the liposomes or on the outside of the liposome vesicles. Chloroform/methanol 2:1 was used in order to break the liposomes shell and release any trapped DNA, this can be used as an indication of the quantity of pDNA inside the liposomes. Agarose gel electrophoresis of cationic lipid:DNA complexes was subsequently used to assess the relative amounts of DNA either free or incorporated into the lipid:DNA complex.

**Figure 3:**
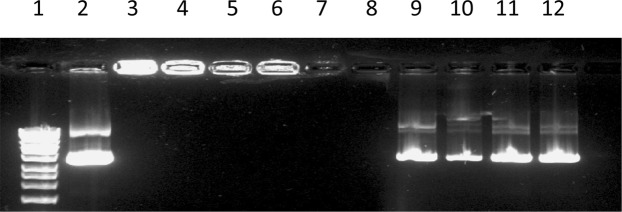
Gel electrophoresis images for nanoAssemblr (microfluidic) prepared liposomes.

**Figure 4:**
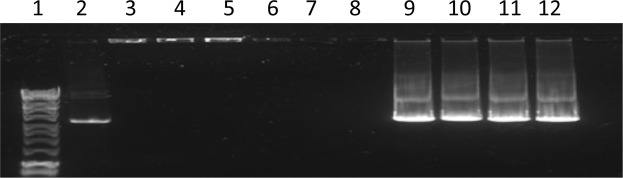
Gel electrophoresis images for Rotary evaporator (thin film hydration) prepared liposomes.

Figures 3 and 4 show that all formulation managed to condense DNA (lanes 3–6) with no DNA migration, as a result of binding and neutralisation of DNA by cationic liposomes. Lanes 9–12 represent lipoplex formulations after liposome shell disruption with chloroform/methanol and subsequent release the pDNA, hence DNA migration seen in lanes 9–12 is as a result of DNA being released from the liposomes following liposome shell disruption.

In Figs 3 and 4 lane 1 is DNA ladder, lane 2 is (pDNA 40 ng/μl), lane 3 is (F2), lane 4 is (F4), lane 5 is (F6), lane 6 is (F8), lanes 7&8 are plain liposomes, lanes 9–12 after breaking lipoplexes (F2, F4, F6, F8, respectively) using 2:1 chloraphorm/methanol. Lanes 3–6 indicated a good encapsulation efficiency of pDNA within liposomes. For formulation composition refer to Table 3 (in experimental section).

**Table 3:** Different liposome formulations prepared by both Rotary evaporator (thin film hydration method) and NanoAssemblr (microfluidic system).

Liposomes formulations	DOPE	DOTAP	CHOLESTERL (CHO)	carboxymethyl-β-cyclodextrin (CD)	Pluronic-F127 (PL)	pDNA
F1 (plain liposome)	8 μmol (5.952 mg)	8 μmol (5.584 mg)	2 μmol (0.77 mg)			
F2 (lipoplex, 1:5 pDNA:liposome)	8 μmol (5.952 mg)	8 μmol (5.584 mg)	2 μmol (0.77 mg)			pDNA
F3 (plain liposome)	8 μmol (5.952 mg)	8 μmol (5.584 mg)	2 μmol (0.77 mg)	3 μmol (3.993 mg)		
F4 (lipoplex, 1:5 pDNA:liposome)	8 μmol (5.952 mg)	8 μmol (5.584 mg)	2 μmol (0.77 mg)	3 μmol (3.993 mg)		pDNA
F5 (plain liposome)	8 μmol(5.952 mg)	8 μmol (5.584)	2 μmol (0.77 mg)	3 μmol (3.993 mg)	0.3μmol (3.78 mg)	
F6 (lipoplex, 1:5 pDNA:liposome)	8 μmol(5.952 mg)	8 μmol (5.584)	2 μmol (0.77 mg)	3 μmol (3.993 mg)	0.3 μmol (3.78 mg)	pDNA
F7 (plain liposome)	8 μmol(5.952 mg)	8 μmol(5.584 mg)	2 μmol (0.77 mg)		0.3 μmol (3.78 mg)	
F8 (lipoplex, 1:5 pDNA:liposome)	8 μmol(5.952 mg)	8 μmol(5.584 mg)	2 μmol (0.77 mg)		0.3 μmol (3.78 mg)	pDNA

**Figure 5:**
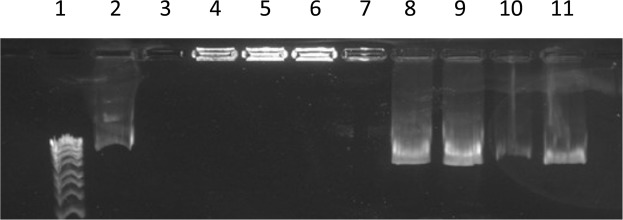
Gel electrophoresis images for nanoAsemblr prepared lipoplexes after the addition of DNase I for 30 min before and after breaking the lipoplexes using chloroform/methanol 2:1 after the addition of DNase I.

**Figure 6:**
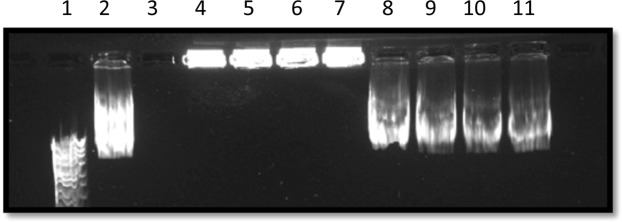
shows Gel electrophoresis images for thin film hydration prepared lipoplexes after the addition DNase I for 30 min before and after breaking the lipoplexes using chloroform/methanol 2:1 after the addition of DNase I.

In order to assess DNA protection and its degradation in the presence of DNase I, using agarose gel electrophoresis of plasmid DNA as a qualitative measure of DNA stability. As shown in Figs 5 and 6 control plasmid DNA was digested by DNase I (lane 2). However all lipoplex formulations demonstrated protection from DNase I, lanes 4–7. After breaking lipoplexes shell and adding DNase I (see lanes 8–11), DNase I was able to digest the condensed pDNA.

In Figs 5 and 6 lane 1 is DNA ladder, lane 2 is (pDNA 40 ng/μl), lane 3 is (empty), lane 4 is (F2), lane 5 is (F4), lane 6 is (F6), lane 7 is (F8), before breaking the lipoplexes. Lanes 8–11 after breaking lipoplexes (F2, F4, F6, F8, respectively) using 2:1 chloraphorm/methanol and adding DNase I. For formulation composition refer to Table 3 (in experimental section).

### Cell transfection

Low transfection efficiency of cationic liposomes is still a major barrier if the aim is to replace the viral vector. The features of liposomes are strictly related to the chemical properties of the cationic and neutral lipids used for their preparations. It is well established that the transfection efficiency can be affected by the composition of the transfection reagent, liposomes size and zeta potential^15,28^ as well as liposomes to DNA ratio^29^. In this study each formulation was tested at four different ratios of DNA: liposomes 1:2, 1:5, 1:10 and 1:20 formulations, this identified the most suitable ratio (1:5) to be used with the aim to reduce any possible toxic effect from liposomal compositions. Multiple, independent (*n* = 3) experiments for each condition to control for biological and methodological variations were used.

The NanoAssemblr method gave the highest encapsulation efficiency, produced homogenised lipoplexes size of about 160 nm, and found to be more practical. Hence, nanoAssemblr produced liposomes were used for transfection optimiation of DNA to liposome ratio.

**Figure 7:**
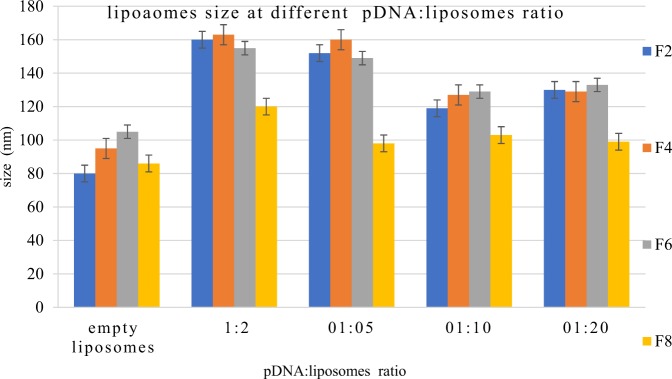
lipoplex size at different ratio of DNA:liposome when prepared by the nanoAssemblr. For formulation composition refer to Table 3 (in experimental section).

Figure 7 shows, the change in liposomes (empty liposomes) size after the addition of pDNA (lipoplexes) at different ratios. Lipoplexes’ size was seen to increase compared to liposomes alone and this is in agreement with many studies.

**Figure 8:**
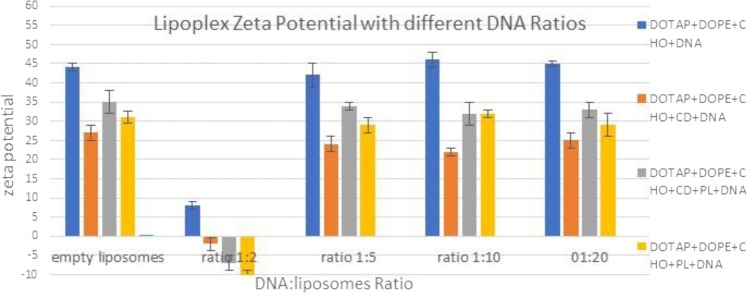
Zeta-potential at different ratio of DNA:liposomes prepared by the nanoAssemblr.

Figure 8 illustrates the zeta potential of liposomes following addition of pDNA to form lipoplexes. In all four different ratios (1:2, 1:5, 1:10 and 1:20 DNA:liposome), at low ratio of 1:2 mostly all formulations became negatively altered. This is due to part neutralisation of the positively charged liposomes by pDNA molecules, which are carrying negative charges. The ratios of 1:5, 1:10 and 1:20 did not alter the original zeta potential of the plain liposomes. Their positively zeta-potential values will not only improve the stablilty of liposomal suspensions by the effect of repulsion but also will enhance the cell uptake, as the cell surface is negatively charged.

**Figure 9:**
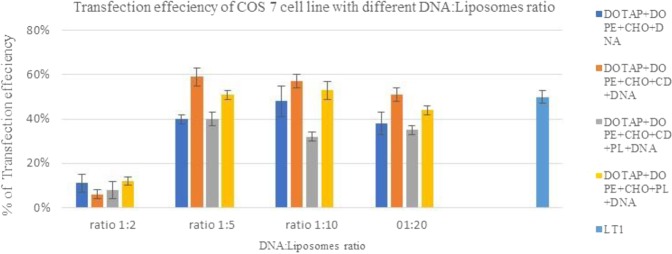
Transfection efficiency of different DNA:liposome ratio and a commercially available liposome reagent, LT1, on COS7 cell line. At ratio 1:5, the effect of caboxy methyl beta cyclodextrin used (CD) was significant compared to Pluronic-F127 ((PL), P < 0.05.

**Figure 10:**
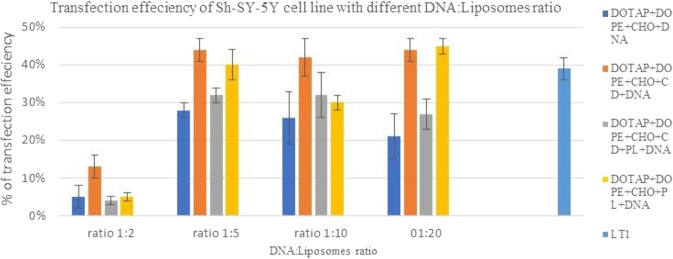
Transfection efficiency of SH-SY5Y cell line with different ratio of DNA:liposomes and a commercially available liposome reagent, LT1. At ratio 1:5, the effect of caboxy methyl beta cyclodextrin used (CD) was significant compared to Pluronic-F127 ((PL), P < 0.05.

Results in Fig. 8 show that zeta potential results were consistent with those of transfection activity (Figs 9 and 10). At low ratio 1:2 there was very low transfection efficiency, this is potentially as a result of the presence of less positively charged lipoplexes. In higher ratios the increased zeta-potential resulted in increased transfection efficiency. Moreover, after ratio of 1:10 (DNA:liposomes) there was increased transfection efficiency with increasing the ratio, as zeta potential became stable (Fig. 8). These results suggested that zeta potential of cationic liposomes had an imporatnat effect on the gene transfection. These results were consistent iwht the findings of Farrow *et al*.^30^ and Wasungu *et al*.^31^ who observed that a positive zeta potential is key in cell transfection.

pDNA must be released into the cytoplasm and transported into the nucleus where transcription takes place.

Liposomes formulations with four ratios of DNA:liposomes were tested on two different cell lines COS 7 and SH-SY5Y. Figures 9 and 10 revealed that all four formulations with ratio of 1:2 had the lowest transfection efficiency. As outlined previously this can be related to the zeta potential, where ratio 1:2 had the lowest zeta potential. At ratio of 1:5 transfection efficiency was optimal and increasing the ration did not lead to increased transfection efficiency.

Figures 9 and 10 showed that the transfection efficiencies of cationic liposomes are cell line specific. COS7 cell line showed a higher efficiency than SH-SY5Y cell line for both prepared lipoplexes and commercially available transfecting liposomes. SH-SY5Y cell line was hard to transfect and showed reistence to liposome cell toxicity at high pDNA:liposome ratios (refer to cell viability section).

Kim *et al*.^32^ stated that a specific cell lines can favor certain lipid compositions, using different ratio of DOTAP:DOPE can help to achieve optimal conditions in gene delivery. It was shown that differences in uptake pathways could affect the intracellular fate of complexes, potentially contributing to the differences in transfection efficiency and this difference is related to the differences in membrane structure. This study has shown that all formulations resulted in higher transfection efficiency in COS 7 than in SH-SY5Y cell lines. This may be explained by Clathrin-dependent endocytosis that accounts for the majority of internalised complexes that penetrated COS7 cells and its limits to particles under 200 nm and hence all prepared formulations are within size of less than 200 nm.

**Figure 11:**
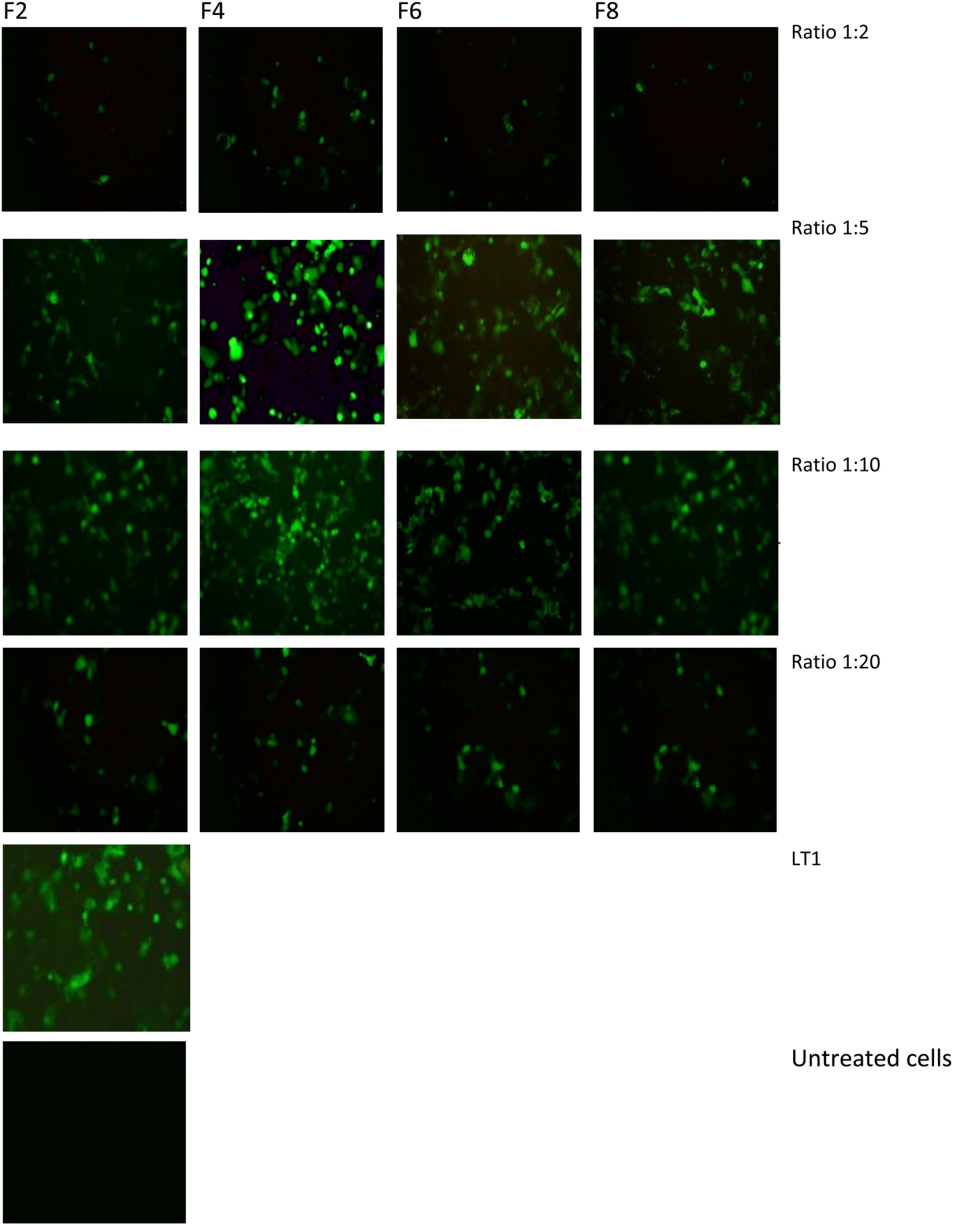
Fluorescence microscopy images (using objective lens of 20×) of transfected COS7 cell line with LT1, commercially available liposome and with different DNA:liposome ratio (1:2, 1:5,1:10 and 1:20) of the four different formulations (F2, F4, F6, F8). For formulation composition refer to Table 3 (in experimental section).

**Figure 12:**
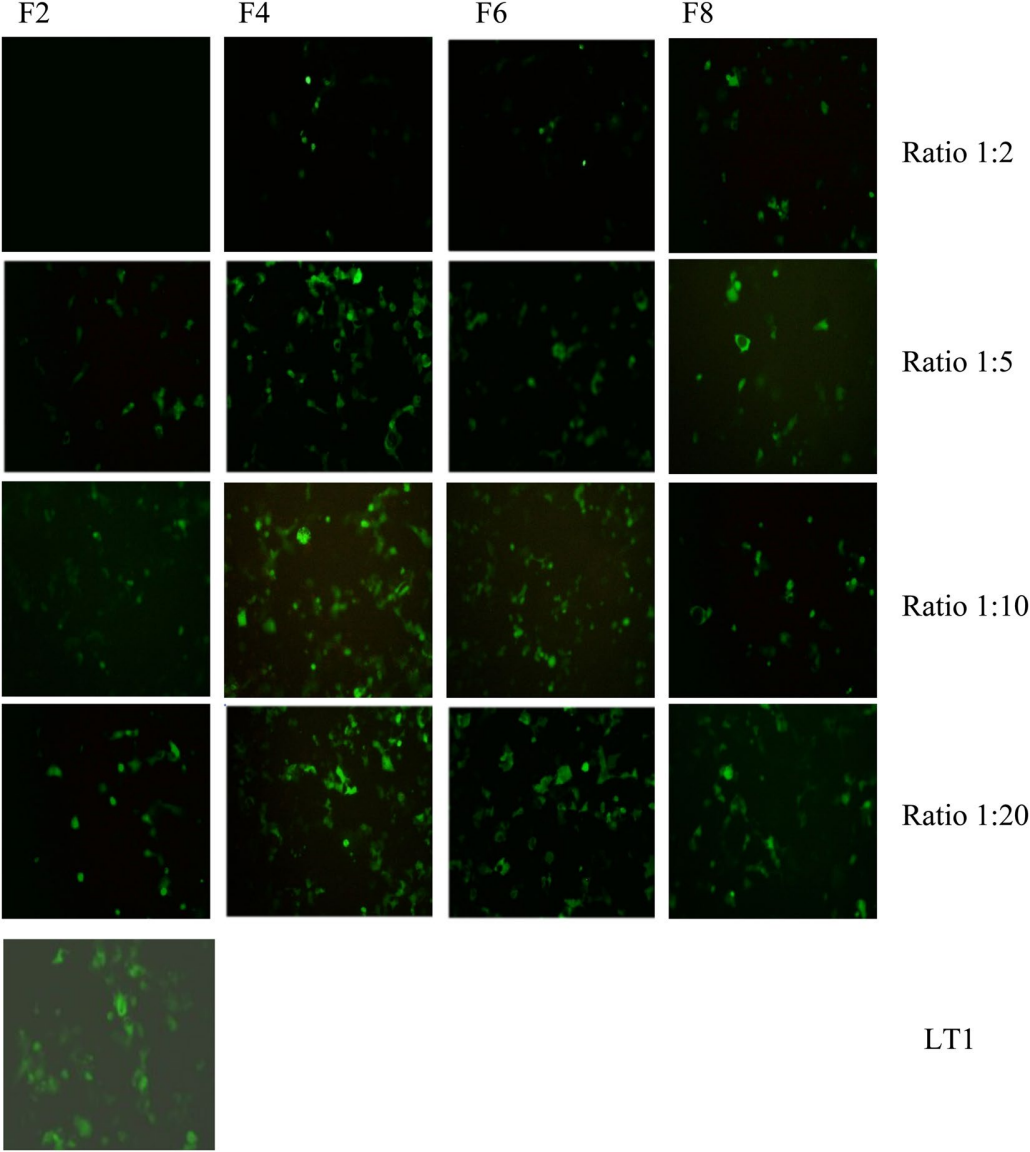
Florescence microscopy images (using objective lens of 20×) of transfected SH-SY5Y with LT1, commercially available liposome and with different DNA:liposomes ratio (1:2, 1:5,1:10 and 1:20) of the four different formulations (F2, F4, F6, F8). For formulation composition refer to Table 3 (in experimental section).

The expression of GFP following the transfection of COS7 and SH-SY5Y cell lines using different lipoplex formulations was assessed with fluorescence microscopy (Figs 11 and 12), compared with commercially available liposomes and quantified by flow cytometry. The highest level of GFP expression was noticed observed after the addition of carboxymethyl -β-cyclodextrin to cationic liposomes (DOTAP, DOPE and cholesterol, F4). The increase in cell transfection after the addition of cyclodextrin could be as a result of reduction in zeta potential which resulted in reduction of aggregation of cationic lipid with protein present in media. Zidovetzki and Levitan^33^ explained that the increase in transfection in the presence of cyclodextrin is assigned to the hydrophobic cavity of cyslodextrin which has the ability to attract cholesterol from the cell membrane. Carboxymethyl -β-cyclodextrin donates cholesterol to cells and by itself causes the efflux of cholesterol from cell membranes, resulting in modulation of the fluidity/rigidity and permeability of the cell membrane. Moreover, the addition of cyclodextrin to cationic lipid has also resulted in an increase of pDNA encapsulation efficiency which might be a reason behind the increase in transfection efficiency. The addition of Pluronic F-127 to cationic liposomes (F6, F8) also improved transfection efficiency over F2.

**Figure 13:**
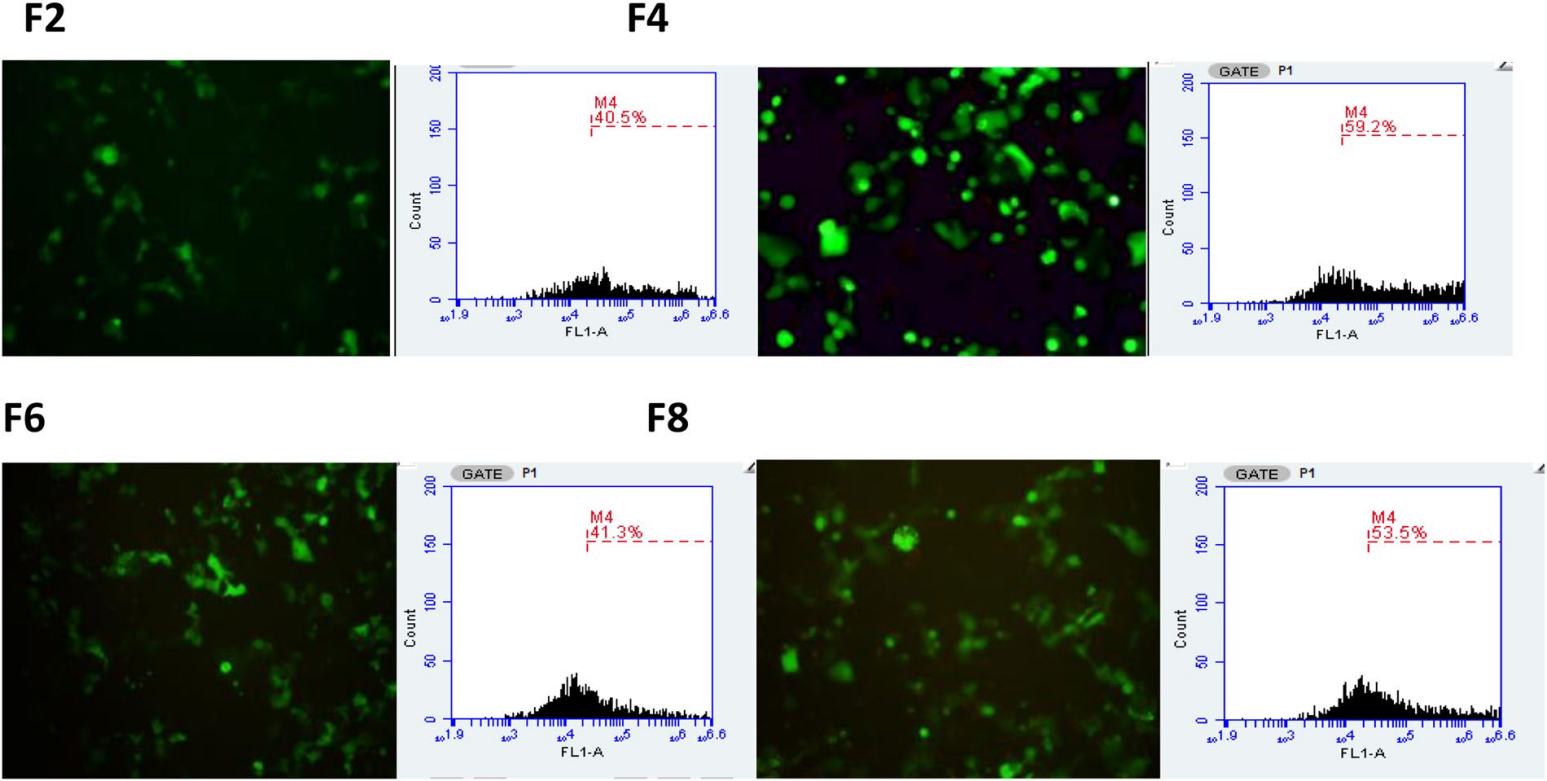
Confocal microscopy images and flow cytometry profiles of COS7 transfection efficiency using Microfluidic method. For formulation composition refer to Table 3 (in experimental section).

**Figure 14:**
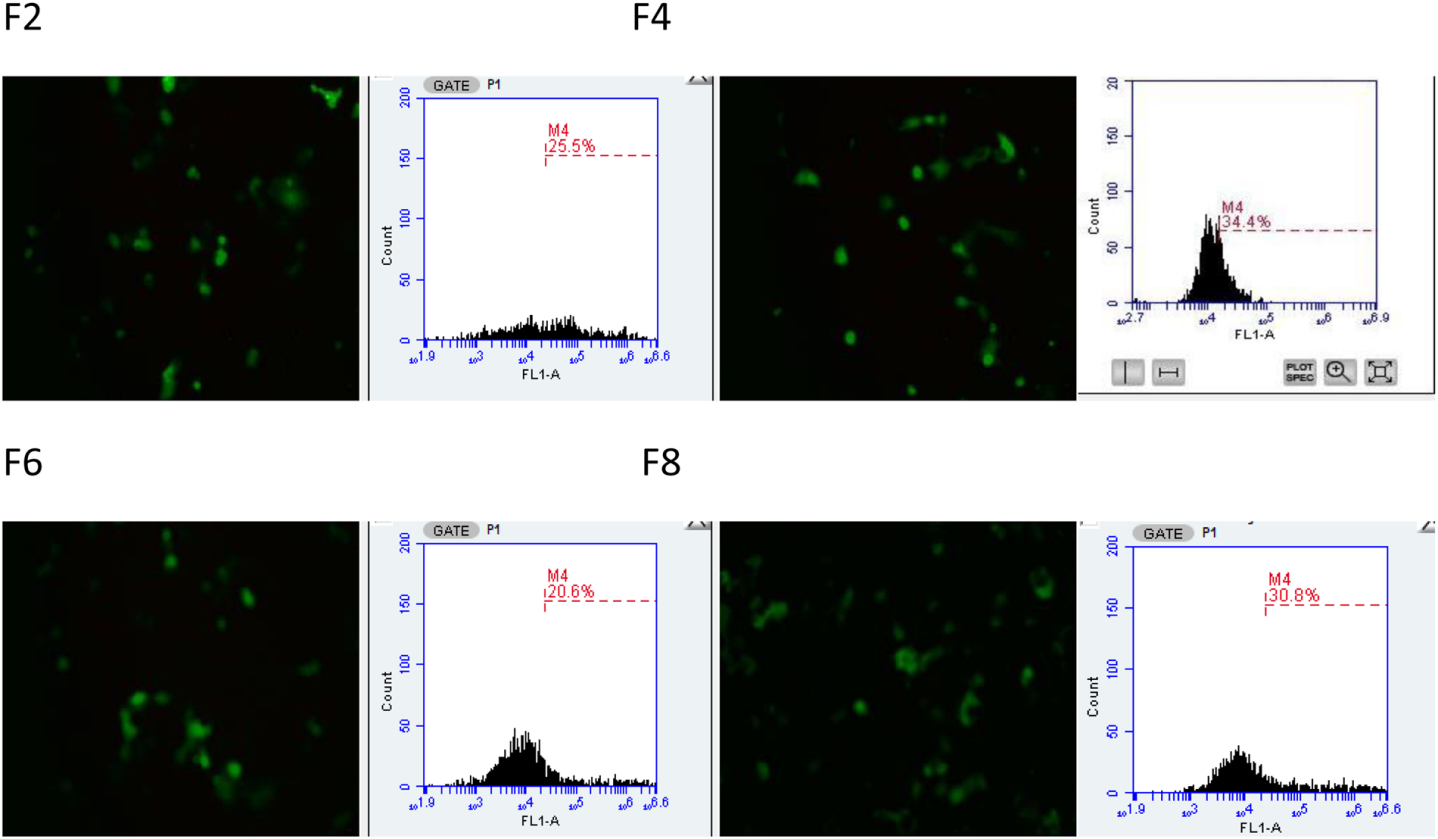
Confocal microscopy images and flow cytometry profiles of COS7 transfection efficiency using Thin Film Hydration method. For formulation composition refer to Table 3 (in experimental section).

Based on the above results ratio 1:5 pDNA:liposome was used to compare the transfection efficiency of the lipoplexes prepared by NanoAssemblr and Rotary Evaporator. Accordingly, results in Figs 13 and 14 have shown that the use of microfluidic system dramatically improved transfection efficiency over thin film hydration method. These results can be explained by that the nanoAssemblr has shown to increase the pDNA encapsulation efficiency and produced smaller and homogenise particle size this was supported by other studies^20,34^. This showed that liposomes with smaller and homogenise size will result in higher transfection efficiency.

### Cell viability

The use of nanoparticles such as liposomes in drug delivery has resulted in reduction of unwanted adverse effect, such as the use of liposomes to deliver doxorubicin has resulted in reduction of cardiac toxicity^35^. In gene delivery, cationic liposomes are the cause of the toxicity. However, in this study DOPA was used in the manufacture of liposomes to reduce their toxicity.

Cationic Liposomes toxicity is mainly due to the positive charge^36^. The head group comprises of primary, secondary, tertiary amines or quaternary ammonium, these positively charged head group may interact with negatively charged components in the cells.

This interaction results on promoting inflammation, cytotoxicity and genotoxicity.

**Figure 15:**
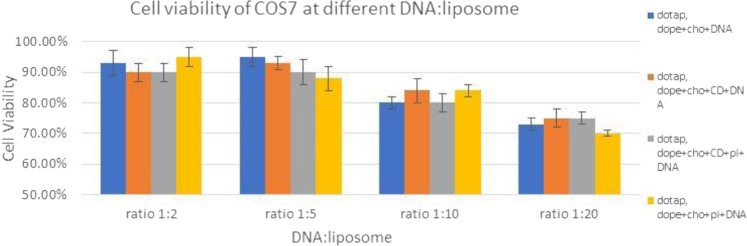
Cell viability of COS7 cell line with different DNA:Liposome Ratio.

**Figure 16:**
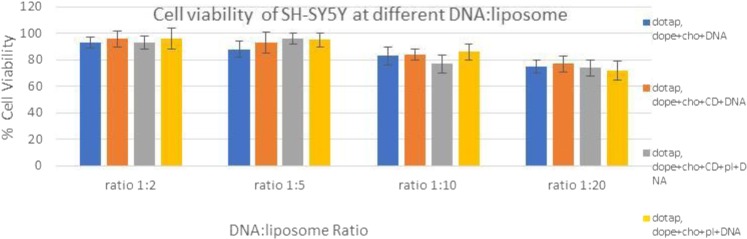
Cell viability of SH-SY5Y cell line with different DNA:Liposome Ratio.

It was reported that the cationic head group within liposomes, and not liposomes themselves, can lead to production of reactive oxygen in lung cells of mice and thus initiating inflammation and toxicity^37^. Due to binding to proteins such as apolipoproteins and immunoglobulins, charged cationic liposomes are easily recognised by reticuloendothelial cells^35^. Many studies have demonstrated that shieling the positively charge will result in reduction of cationic liposomes toxicity. Results shown in Figs 15 and 16. Ratios 1:10 and 1:20 which have the higher liposomes ratio have resulted in higher cell toxicity due to higher lipid contents; the cell viability was independent on the addition of CD and PL based on this study. These results therefore are consistnet with the theory of cationic liposomes in that increasing the lipid concentration will lead to increasing liposomes cytotoxicity^38^.

### Liposome formulations stability in PBS buffer, pH 7.4

Liposomes’ size was monitored weekly during storage for 12 weeks at 4 °C and 37 °C. Aqueous liposomal suspensions in PBS, pH 7.4, prepared by thin film hydration and microfluidic methods were characterized in tripilicate.

**Figure 17:**
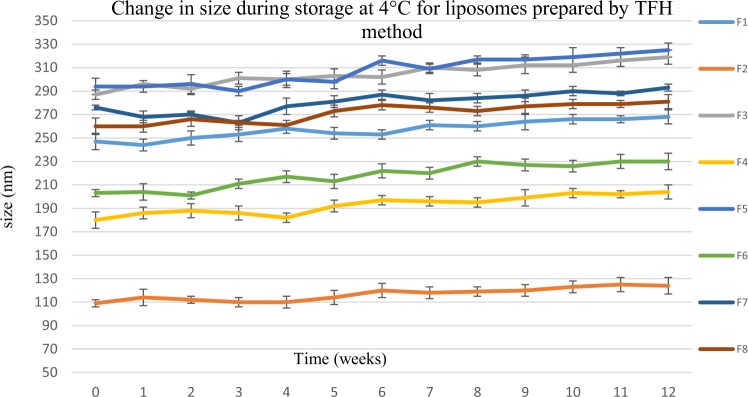
The change in liposomes’ size over 12 weeks storage at 4 °C. Liposomes were prepared using thin film hydration (TFH) method, for formulations’ composition refer to Table 3 (in experimental section).

**Figure 18:**
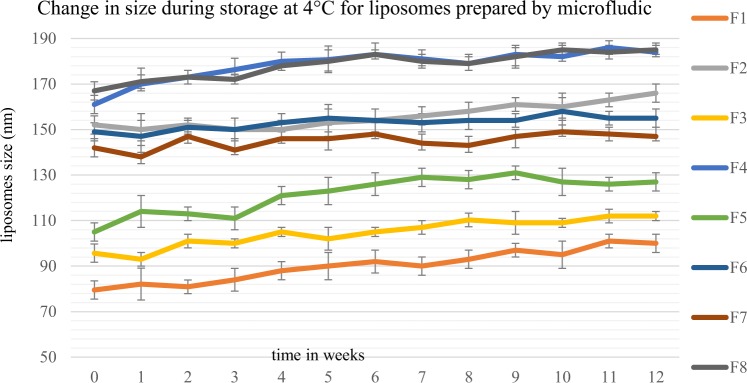
The change in liposomes’ size over 12 weeks storage at 4 °C. Liposomes were prepared using microfluidic method, for formulations’ compostion refer to Table 3 (in experimental section).

Results in Figs 17 and 18 showed that all formulations prepared by microfluidic and thin film hydration methods were stable at 4 °C, as there was insignificant change in particle size, (p > 0.05). This was in agreement with a study by Zuidam and Crommelin^39^.

**Figure 19:**
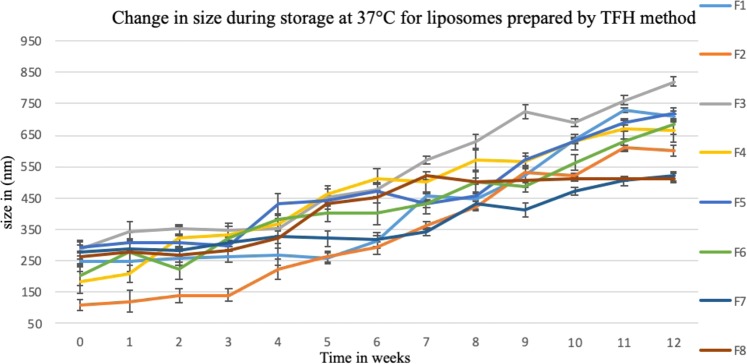
The change in liposomes’ size over 12 weeks storage at 37 °C. Liposomes were prepared using thin film hydration (TFH) method, for formulations’ compostion refer to Table 3 (in experimental section).

**Figure 20:**
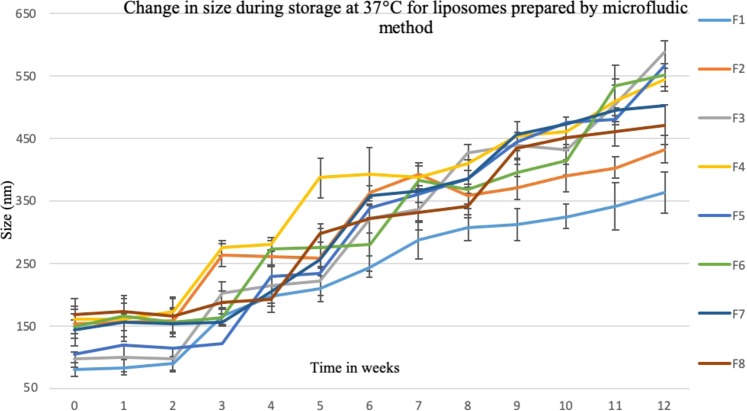
The change in liposomes’ size over 12 weeks storage at 37 °C. Liposomes were prepared using microfluidic method, for formulations’ compostion refer to Table 3 (in experimental section).

At 37 °C, all formulations have increased in size (Figs 19 and 20). However, liposomes prepared using microfluidic method showed much smaller change in particle size compared with those prepared by the thin film hydration method. This can be explained by the fact that NanoAssemblr produced smaller and more homogenised size distribution when compare to the thin film hydration method. These results were in line with that by Kastner *et al*.^40^ who compared the stability of liposomes (Egg phosphatidylcholine (PC) and cholesterol) prepared using microfluidic and lipid hydration techniques. They reported that liposomes were stable under 4 °C for 60 days. However at 40 °C liposomes lost their structures and contents; also liposomes prepared by microfluidic method were much more stable than liposomes prepared by lipid hydration method. Hence, it is recommended that liposomal colloidal suspensions should be stored at low temperatures, 4 °C or less.

## Conclusion

In conclusion, the incorporation of carboxymethyl -β-cyclodextrin with cationic lipid has shown to improve encapsulation efficiency of pDNA, as well as transfection efficiency and cell viability, with and without the addition of Pluronic F-127. The NanoAssembler method showed to produce homogenies size, low PDI and increased the pDNA encapsulation efficiency. It also produced smaller liposomes with uniform profiles which are less succeptible to aggregation. Moreover, this work has demonstrated the use of microfluidic hydrodynamic flow focusing (HFF) method and its advantage over the rotary evaporator (thin film hydration method), which HFF has the ability to control size in one step.

## Experimental Section

### Materials

DOPE (1,2-dioleoyl-sn-glycero-3-phospho ethanol amine and DOTAP (1,2-dioleoyl-3-trimethylammonium-propane) were purchased from Lipoid (Germany). Carboxymethyl-β-cyclodextrin, Pluronic-F12, cholesterol, DMEM, Fetal bovine serum (FCS), L- Glutamine and Opti-MEM I Reduced-Serum Medium were purchased from Sigma Aldrich (UK). pc DNA3.1-GFP and maxi prep kit were purchased from thermofisher scientific, UK, COS7 and SH-SY5Y cell lines were obtained from ATCC, UK. *E coli*, ampicillin containing agar plated, Luria-Bertaini and agarose were also provided by University of Sunderland. TransIT-LT1 liposomal transfection reagents were purchased from Mirus Bio LLC Madison, (USA), Promega QuantiFluor® ONE dsDNA System was purchased from Promega (UK).

### Plasmid DNA preparation

In this study, pDNA expressing green fluorescent protein (pc DNA3.1-GFP) was amplified by transformation of *E. coli* to produce large quantity of the plasmid. Cells were plated into the ampicillin containing agar plates and stored at 37 °C overnight. One colony was picked from the plate and placed into 100 mls of LB (Luria-Bertaini) medium and left for 48 hours on the shaker. Following this, the medium was purified using a Maxiprep kit. following manufacture protocol (Invitrogen, UK). Purity and quantity of the plasmid were checked using NanoDrop lite (themo, UK) purity was 1.9 and quantity was diluted to produce 1 μg/μl using TE buffer. This was also confirmed by taking UV measurement at 260 nm and 280 nm wavelengths.

### Liposomes preparation by thin film hydration method

DOTAP, DOPE and cholesterol with a molar ratio of 8:8:2 (Table 3) were dissolved in round flask glass, with 2 mls of ethanol. The solvents were evaporated over two hours at 60 °C using the rotary evaporate pressure set at 465 mbar. Liquid nitrogen applied to dry any left-over solvent. Pluronic F127 and Carboxymethyl-β-cyclodextrin were dissolved in distilled water at concentration of 4 mg/ml.

The lipid was then rehydrated using an aqueous medium (distilled water or carboxymethyl-β-cyclodextrin in distilled water or Pluronic F127 or carboxymethyl-β-cyclodextrin in distilled water or Pluronic in distilled water) to produce final lipid concentration of 10 mg/ml (see Table 3 for more details). The mixture was then vortexed for 2 min and ultrasonic bath sonication for 20 minutes to produce plain liposomes. Lipoplexes (liposomes with pDNA) were prepared by adding the required amount of pDNA (at a concentration of 1 mg/ml) to 1 ml of each liposome formulation (at a lipid concentration of 1 mg/ml). For example to prepare lipoplexes with the used ratio, 1:5 ratio, of pDNA:Liposome, 200 microlitre of pDNAwas added to 1000 microlitre of the prepared liposome.

### Liposomes preparation by microfluidic method

DOTAP, DOPE and cholesterol were dissolved in 1 ml ethanol with a molar ratio of 8:8:2 (see Table 3), this ration has been chosen, based on preliminary studies, as it gave a good transfection efficiency. The ethanol-lipid solution was injected into the first inlet. The aqueous phase was injected with 3 ml of distilled water contained carboxymethyl-β-cyclodextrin; Pluronic F-127 and carboxymethyl-β-cyclodextrin; or Pluronic F-127 alone (Table 3). Aqueous dispersions of the liposomes were collected from the outlet, resulting from the mixing of two adjacent streams and centrifuged at 13000 rpm for 40 minutes to remove the ethanol resides. Then, re-suspended in distilled water to make up a concentration of 10 mg/ml.

The formed liposomes were used to prepare the lipoplexes (1:5 ratio of pDNA:Liposome) as above.

In order to optimise liposomes size and zeta-potential, the NanoAssemblr was run at different|: flow rate ratio (FRR) between the lipid and water (at 1:0.5, 1:1,1:3 and 1:5) and the total flow rate (TFR), at 12 ml/min, 9 ml/min, 5 ml/min and 2 ml/min.

### Particle size, zeta potential, polydispersity and transmission electron microscopy

Liposomes size and zeta potential are important characteristics of liposomes formulations especially in gene delivery. Negatively charged particles can be rapidly opsonised and massively cleared by fixed macrophages of the reticuloendothelial system (RES) in the blood stream. Dynamic light scattering (DLS) technique was used to report the intensity mean diameter (z-average) and the polydispersity (PDI) of all liposome formulations (Malvern Zetasizer Nano-ZS (Malvern Instruments, Worcs., UK)). DLS measures the size of liposomes suspended in distilled water. Transmission electron microscopy was applied to study the morphology of the prepared liposomes.

### Encapsulation efficiency

Encapsulation efficiency of pDNA was measured using the NanoDrop lite. 30 μl of pDNA at concentration of 1 μg/μl was added to 60 μl of liposomes at concentration of 10 mg/ml to give ratio of 1:20 DNA:Liposomes of each liposome preparation, vortex for 3 second and left at room temperature (20 °C) for 20 minutes. Samples were then centrifuged for 45 min at 13000 at 4 °C. The supernatant was separated from the pellets. The reading for pDNA concentration in the supernatant was taken and subtract from the total quantity of pDNA (see Eq. 1). The pellets were then broken by a chloroform/methanol 2:1 to extract the pDNA. 400 μl of chloroform/methanol 2:1 was added to the pellet and vortexed until all lipid dissolved. 100 μl of distilled water was added to the mixture and centrifuged for 10 minutes. The aqueous layer was used to quantify the encapsulated pDNA by measuring its UV absorption at 260 nm.

In order to confirm the results. Promega QuantiFluor® ONE dsDNA System was used by following the manufacturer protocol. Briefly a serial of DNA concentrations was prepared from 25 ng/μl to 400 ng/μl, this will be used as standards. Using 96 plates, 200/μl of the QuantiFluor® ONE dsDNA Dye was added to each well including standard and blank. 1 μl of each DNA dilution was added to the dye in triplicate. labelled Standards A–G. For the blank, 1 μl of 1X TE buffer were pipetted into row H in triplicate. 1 μl of each formulation (F2, F4, F6 and F8 samples were added to A,B,C and D rows in triplicate. Fluorescence was measured at (504nmEx/531nmEm), using TriStar LB 941 Microplate Reader (Berthold Technology). To calculate pDNA concentration, fluorescence of the blank sample (1X TE Buffer) was subtracted from each standard and sample. Using the data from DNA standards to generate a standard curve of fluorescence versus DNA concentration, concentration of DNA in each formulation was caluclated.

1 $$\begin{array}{c} \% \,{\rm{Encapsulation}}\,{\rm{efficiency}}\,\\ =\,\frac{({\rm{theoretical}}\,{\rm{DNA}}\,{\rm{concentration}}-{\rm{pDNA}}\,{\rm{concentration}}\,{\rm{in}}\,{\rm{supernatant}})\times {\rm{100}}}{{\rm{Theoreticalp}}\,{\rm{DNA}}\,{\rm{concentration}}}\end{array}$$

### Gel-electrophoresis: Protection of pDNA against DNase I

Lipoplexes were assessed against DNase I degradation as follows:

**1***.* adding 2 units of DNase I to each intact lipoplex formulation containing 1 μg pDNA and 20 μg liposomes/100 μl buffer, to give ratio of 1:20 DNA:liposomes, at 37 °C for 30 min. 10 μL from each sample was run in gel electrophoresis to measure the protection that liposomes provide for pDNA. This can also be used to measure if pDNA has been encapsulated inside the liposomes, or is just bound to the liposome membrane.

**2**. Adding DNase I to the dissociated lipoplexes (20 μl of chloraphorm/methanol 2:1 were used in order to dissociate the lipid/DNA complexes before adding the DNase I). After 30 min, 10 μL from each sample was run in gel electrophoresis

**3**. 1 μg of pDNA was incubated with 2 units of DNase I in 100 μl buffer (this was used as a reference to compare with intact lipoplex formulations).

All samples run in 1% agarose gel for 60 min at 90 vm. Results were analysed by UV trans-illumiator with digital imaging (BioRad Laboratories, Inc).

### Transfection efficiency

The efficiency of each liposome formulation was measured, by transfecting pDNA3.1-GFP to COS7 and SH-SY5Y cell lines. 24 hrs before transfection, 3 × 10^5^ of both cell lines were plated in 6 well plates with 2.5 mls of DMEM, 10% fetal bovine serum (FCS) and 1% L- Glutamine, at 37 °C and 5% CO_2_. Cells were ≥70% confluence. Lipoplexes were prepared by diluting 2 μg of pDNA in 250 μl Opti-MEM I Reduced-Serum Medium. Although lipoplexes 1:5 pDNA:liposome was used for all characterisation but the selection of this ratio was based on testing the effect of different ratios on transfection efficiency. Hence, the required ratios of pDNA to liposomes (1:2,1:5, 1:10, 1:20) were prepared and incubated at room temperature 20 °C for 30 minutes. After incubation lipoplexes were added to each cell well drop-wise to different areas of the wells. Transfected cells were placed back in the incubator at 37 °C and 5% CO_2_, for 48 hours and then processed for flow cytometry, FACS and fluorescence microscopic analysis. TransIT-LT1 liposomal reagent was used as a positive reference and untreated cell as a negative reference. To quantify transfection efficiency, EGFP positive cells were measured using FACS, BD Accuri C6 plus (Becton Dickinson Bioscience, USA). Firstly, cells were washed with phosphate buffer saline (PBS) and in order to detach cells from plates 200 μl trypsin was added, and cells were incubated for 5 minutes. Cells were suspended in 1 ml of media and centrifuged at 400 G for 5 minutes, followed by removing the supernatant. Then celles were re-suspended in 1 ml PBS and taken to FACS for quantification of GFP. Negative Control samples (non-transfected cells) were displayed on a forward scatter (FSC) versus side scatter (SSC) dot plot to establish a collection gate and exclude cells debris. Cells transfected with TransIT-LT1 reagent, were used as a positive control sample. Transfection efficiency was expressed as the percentage of EGFP positive cells at 525 nm (FL1) after excluding dead cells. For each sample 10,000 events were collected. Each formulation was analysed in triplicate.

### Cell viability

Cell viability was evaluated using Propidium iodide (PI) and 3-(4,5-Dimethylthiazol-2-Yl)-2,5-Diphenyltetrazolium Bromide (MTT) test. Different pDNA:liposomes were tried to see their effect on cell viability whether is due to plain liposomes’ toxicity.

Propidium iodide (PI), this dye binds to double stranded DNA by intercalating between base pairs. PI is excited at 488 nm, and with a relatively large Stokes shift, emits at a maximum wavelength of 617 nm. 5 μL of PI was added to each sample including positive and negative control samples. The fluorescent signal corresponding to dead cells was measured at 650 nm (FL2).

Cell Proliferation Kit I (MTT) from Sigma-Aldrich, UK, was used: MTT 3-(4,5-dymethyl thiazol 2-y1)-2,5-diphenyl tetrazolium bromide (MTT, mitochondrial respiration analysis) following the manufacture protocol. Briefly, the assay is based on the cleavage of the tetrazolium salt MTT in the presence of an electron-coupling reagent to produce water-insoluble formazan salt^41^. The formazan dye is quantitated using a scanning multi-well spectrophotometer (TriStar LB 941 Multimode Microplate Reader, Berthold Technologies GmbH & Co). The measured absorbance directly correlates to the number of viable cells. Cells were seeded on 96-well plate (20,000 cells/well). 24 hrs after seeding, cells were treated with 1.25 μg of each plain liposome and lipoplex formulation and incubated again for a 48 hrs period. Then, 0.01 ml (from a final concentration of 0.5 mg/ml) MTT was added to each well. Then, after 4 hours of incubation at 37 °C, isopropanol with 0.04 N HCl was added. The isopropanol dissolves the formazan to give a homogeneous blue solution suitable for absorbance measurement. The absorbance of each well was measured at 570 nm using the Microplate Reader (TriStar LB 941, Berthold Technology GmbH & Co). Viability was calculated and expressed as a percentage of the positive control (i.e. untreated cells), Eq. 2:

2 $$ \% \,{\rm{Cell}}\,{\rm{viability}}=(\frac{{\rm{Absorbance}}\,{\rm{of}}\,{\rm{treated}}\,{\rm{cell}}}{{\rm{Absorbance}}\,{\rm{of}}\,{\rm{untreated}}\,{\rm{cell}}})\times 100$$

### Storage stability

Sizes of all prepared liposomes and lipoplexes were investigated in PBS aqueous medium (pH 7.4) for vesicles’ stability at 4 °C (using a fridge) and at 37 °C (using an oven); samples were stored for 12 weeks. The samples were measured, weekly, in triplicate.

### Statistical analysis

All measurements were replicated at least three times. The results were evaluated statistically with SPSS software. Univariate analysis of variance was used as statistical analysis. Levene test was used to test the sample has equal variances. Equal variances cross sample is called homogeneity of variance. Tukey test was used for normal distribution. The data are considered significant if P < 0.05. All data were stated as mean ± standard deviation.
